# Laminar population analysis of multielectrode recordings from rat primary auditory cortex

**DOI:** 10.1186/1471-2202-12-S1-P87

**Published:** 2011-07-18

**Authors:** Eivind S Norheim, Francois D Szymanski, Klas H Pettersen, Ulf G Indahl, Jan WH Schnupp, Gaute T Einevoll

**Affiliations:** 1 Department of Mathematical Sciences and Technology, Norwegian University of Life Sciences, Ås, Norway; 2 Robotics, Brain, and Cognitive Sciences Department, Italian Institute of Technology, Genova, Italy; 3 Department of Physiology, Anatomy and Genetics, University of Oxford, Oxford, UK

## 

The technology for large-scale electrical recordings is rapidly improving, and extracellular recordings with multielectrode arrays now offer a unique window into neural activity at the population level. The high-frequency part of the recorded signal (MUA; multi-unit activity) is a measure of action-potential firing of neurons in the immediate vicinity of the electrode contacts, while the low-frequency part (LFP; local-field potential) appears to mainly be a measure of subthreshold dendritic activity of surrounding neurons [1,2]. The interpretation of MUA and LFP signals in terms of neural circuit activity is not straightforward, however, and new analysis methods are needed to take full advantage of the technological developments.

*Laminar population analysis* (LPA) [1] was introduced a few years ago as a new method for extracting information about population activity. Based on physiologically-constrained joint modeling of the MUA and LFP signals, both the (i) laminar organization and time-resolved firing rates of the main cortical populations, and (ii) the functional synaptic connection patterns between these populations, were estimated. Later, the population firing-rates extracted by means of LPA were used to specify mathematical neural-network models describing thalamocortical and intracortical connections between neural populations in the rat somatosensory (barrel) system [3].

In Ref. [1] LPA was used to analyze stimulus-averaged laminar (linear) electrode data from the rat barrel system, and the predicted time-resolved population firing rates and synaptic connection patterns were found to be in qualitative agreement with previous experimental findings. Here we apply LPA to recently recorded laminar-electrode data from the primary auditory cortex of rats [4]. In this study the rats were exposed to a large variety of different auditory stimuli to investigate the phenomenon known as ‘stimulus-specific adaptation’, i.e., that neurons respond stronger to unexpected stimuli. Such rich data sets where the cortical neural circuits of interest potentially exhibit a large variety of activation patterns, are particularly well suited for analysis by means of LPA. Results from LPA analysis will be presented, highlighting the differences and similarities seen between the sensory-evoked cortical response patterns in primary auditory and somatosensory cortices of rats.
